# Analysis on Effects of Joint Spacing on the Performance of Jointed Plain Concrete Pavements Based on Long-Term Pavement Performance Database

**DOI:** 10.3390/ma15228132

**Published:** 2022-11-16

**Authors:** Jiaqing Wang, Xiaojuan Luo, Xin Huang, Yao Ye, Sihan Ruan

**Affiliations:** 1 College of Civil Engineering, Nanjing Forestry University, Nanjing 210037, China; 2 Key Laboratory of RC&PC Structures of Ministry of Education, Southeast University, Nanjing 210096, China; 3 School of Civil Engineering, Southeast University, Nanjing 210096, China

**Keywords:** jointed plain concrete pavement (JPCP), long-term pavement performance (LTPP), joint spacing, pavement distresses, optimum design

## Abstract

With the day–night temperature and moisture levels changing every day, expansion and shrinkage of concrete slabs is always occurring; therefore, joints provide extra room for concrete slab deformation. The joint spacing in jointed plain concrete pavement (JPCP) is continuously affecting long-term pavement behaviors. In this study, data from the Long-Term Pavement Performance (LTPP) program were analyzed, and the behaviors of JPCP with different joint spacings were compared to discover the joint spacing effects. Since LTPP has an enormous database, three representative sections located in different states were selected for analysis, where the variable factors such as temperature, moisture, and average annual daily truck traffic (AADTT) were almost the same between the three sections. Three different joint spacings, including 15 ft (4.5 m), 20 ft (6 m), and 25 ft (7.5 m), were compared based on the collected LTPP data. The involved long-term pavement performances, such as average transverse cracking (count), average JPCP faulting, international roughness index (IRI), and falling weight deflectometer (FWD) deflections were compared between JPCP with different joint spacings. Based on the comparative analysis, the JPCP constructed with a 15 ft joint spacing demonstrated the best long-term performance. It showed no transverse cracking, the lowest average JPCP faulting, the best IRI value, and the smallest FWD deflection during the entire in-service period. With proper joint spacing, the cost of road maintenance throughout the life cycle could be significantly reduced due to there being less distress. Therefore, it is recommended to optimize the joint spacing to about 15 ft in JPCP in future applications.

## 1. Introduction

As one of the most frequently used construction materials, concrete is widely used in the construction and civil engineering industries. The first concrete road was built in Bellefontaine, USA in 1893 and is still in use. In response to the national green energy-saving policy, the civil engineering industry has been improving the performance of concrete in recent years to achieve the purpose of both reducing the amount of concrete used and meeting the requirements of building use. Concrete is made of portland cement, aggregates, and water, which are restricted to rigid pavements. There are five main types of concrete pavement: jointed plain concrete pavement (JPCP), jointed reinforced concrete pavement (JRCP), continuously reinforced pavement (CRCP), permeable concrete pavement, and compacted concrete pavement (RCC). Transverse cracks are cracks that are perpendicular to roadway alignment. These are generally due to temperature changes with shrinkage during curing. If there is no room for concrete to expand or contract, the concrete will crack. In fact, all transverse cracking in concrete pavements is due to tension within the slabs, and it can be very difficult to predict where the cracking will occur. In order to avoid transverse cracks, contraction joints have been constructed. JPCP is the original type of rigid pavement, constructed of closely spaced plain concrete without reinforcement, divided by joints to resist the development of cracks. It is the most common type of concrete pavement in the U.S. and Canadian highway systems. Rigid pavements transfer vehicle loads generated during vehicle travel through joints, and relative joint depths of less than 25% can affect joint load efficiency [1]. The load transfer efficiency (LTE) is provided by the joint, the dowel bar, or the aggregate interlocking structure that can be used for load transfer through the joint, where the aggregate interlocking structure is supported by the joint, the dowel bar, the base, and the concrete shoulder. Lack of aggregate interlocking and reduced shear resistance along the joints can lead to reduced LTE, while LTE is also affected by erosion in the dowel bar [2]. Sadeghi and Hesami [3] found that increasing the modulus of elasticity or thickness of the concrete slab base can increase the efficiency of load transfer, reduce the deflection of joints, and reduce damage to pavement joints. Dowel bars can provide uniform deflection of the slab when subjected to moving axial loads [4]. H.B. Sii et al. [5] analyzed dowel bar loosening on JPCP based on a three-dimensional finite element and demonstrated that the presence of gaps between dowel bars and slabs leads to increased stresses in the subgrade and a significant reduction in LTE. The use of force transfer bars is an effective method to control joint fracture, and Davids et al. [6] proposed a force transfer bar modeling technique that allows explicit modeling of force transfer bar loosening. This technique found that the principal tensile stresses in the concrete slab increased significantly and the vertical stresses in the subgrade increased under wheel and temperature loads when the distance between the dowel bar and the concrete slab was lower than 0.24 mm. Without joints, transverse and longitudinal cracking would form in natural directions and locations, and once those cracks formed, the concrete pavement would be damaged quickly under repeated load, temperature, and moisture changes. Joints have the most considerable effects on the performance of plain concrete pavements. In addition to considering the single joint performance, some scholars have also studied the long-term performance of the JPCP structures.

The Long-Term Pavement Performance (LTPP) program is the largest pavement investigation conducted to date and is an important source of monitoring the road performance information for the pavement system. The program monitors more than 2500 test sections of operational highways in the United States and Canada and collects data through a partnership between highway agencies and LTPP program organizations. Malla et al. [7] constructed a resilient modulus prediction model using data extracted from the LTPP database, and Dong et al. [8] analyzed the effect of different factors on asphalt pavement cracking based on LTPP data. Gong et al. [9] illustrated that the LTPP database is vital and can be used to analyze the structural performance of a wide range of pavements. The JPCP performance was also studied based on LTPP data. For traffic safety and to ensure pavement performance, JPCP needs to be maintained and rehabilitated during its service life. JPCP health assessments are based on conditions such as plate cracking direction, length, and width, which can lead to more accurate maintenance decisions by referring to LTPP data. Wang et al. [10] analyzed the LTPP cracking data to explore the relationship between fatigue failure points and various influencing factors, and the results demonstrated that an increase in traffic volume and precipitation intensity, and the number of freeze–thaw cycles, will reduce the fatigue life of the JPCP. Additionally, a survival model that can be used to predict the pavement condition was also proposed based on the collected LTPP data. Other research also found that short-slab JPCP can be used to reduce crack width, enhance aggregate interlocking, and reduce pavement deterioration [11]. The main form of JPCP structural damage is fatigue cracking, and Kim et al. [12] believed that fatigue cracking spreading from the bottom to the top is not the only formation possible; in a critical condition, cracking could also develop from the top to bottom. Having optimum joints in the pavement can guide the cracking direction based on the research findings. Meanwhile, other factors such as temperature, humidity, slab thickness, and subgrade design also affect the long-term performance of infrastructures [13,14]. Buckling and warping can affect the structural behavior of the concrete pavement, and higher temperatures can affect the connection at the joints and cause the concrete to warp. Without joints, transverse and longitudinal cracks will form in the deformation of concrete slabs. Once these cracks are formed, concrete pavements can be damaged rapidly under repeated wheel loads and changes in the environment, while lower humidity can also cause excessive upward warping of the slab [15]. With changing temperatures, thermal stresses are generated inside the concrete slabs, while the heat of cement hydration and evaporation from the pavement surface also greatly influences the internal temperature and humidity [16]. Asbahan et al. [17] found experimentally that temperature and humidity in concrete slabs caused the slab to curl upward and produced cracks that developed from top to bottom. The total curvature of the restrained slab was also higher than that of the unrestrained slab. Shafiee et al. [18] found that the combination of high-temperature weather, high wind speed, and low relative humidity produced a large internal temperature gradient in concrete slabs, which reduced the strength and durability of JPCP. Besides the surface layer of JPCP, granular and stabilized subgrades are often used in the design of JPCP structures, and the stabilized subgrade is used more frequently. Mu et al. [15] evaluated the AASHTO pavement M–E design guidelines and found that the M–E design overestimated the stresses in the unbonded subgrade while underestimating the stresses in bonded subgrade when the environmental effect is obvious. It also underestimated transverse cracking in JPCP with stabilized subgrade while overestimating that with the aggregate subgrade. In addition to the structure design effects, different types of concrete materials also show different effects on the concrete structures [19,20]. Shi et al. [21,22] studied the utilization of RAP instead of coarse aggregate in concrete pavement materials. The results showed that the application of RAP decreased the tensile strength of JPCP, increased the stress concentration, and led to an increased chance of transverse cracking. Conventional plain concrete (PC) leads to a large design thickness of concrete slabs, and different fiber-reinforced concrete (FRC) materials are used in JPCP to reduce the slab thickness. Ali et al. [23] found that JPCP constructed using FRC with glass fiber and polypropylene (PP) fiber was more economical and environmentally friendly than the JPCP produced conventionally. Colley et al. [24] found that when the load, slab thickness, and subgrade were kept constant, the joint effectiveness was reduced with the increased crack width. With a constant crack width, the joint effectiveness decreased with the application of load, but with the increase in the number of loading cycles, the rate of decrease became slower. It can be summarized that no matter the changes in structure designs and material types, the joints are the most important influencing factor in the performance of JPCP. Based on the current research, it was found that few studies have been conducted on the effect of joint spacing on JPCP performance, which could also be a dominant factor that affects the long-term performance. The joint spacing is also a construction related issue when designing a JPCP structure. The presence of joints cannot be dismissed with regard to the limitations of natural cracks and the expansion/shrinkage of concrete materials [25]. The range of joint spacing is usually between 15 ft (4.5 m) and 30 ft (9 m), but the optimum joint spacing is not clear when considering the long-term performance.

This research is based on the LTPP database. In order to find the effects of joint spacing on the JPCP performance, three different joint spacings (15 ft (4.5 m), 20 ft (6 m), and 25 ft (7.5 m)) were selected and compared in terms of the extracted LTPP data collection. Not only annual average daily truck traffic (AADTT) was chosen as a constant, with a level of 3000~6000, but also the climate situation was controlled to make sure the only variable value was the joint spacing. Based on the extracted LTPP data, the transverse cracking, faulting, IRI, and FWD deflection during the service periods were compared to the long-term behaviors of JPCP with various joint spacings. The findings from this study could contribute to the future application of JPCP with performance-optimized joint spacing.

## 2. Collection and Extraction of the LTPP Database

**Figure 1:**
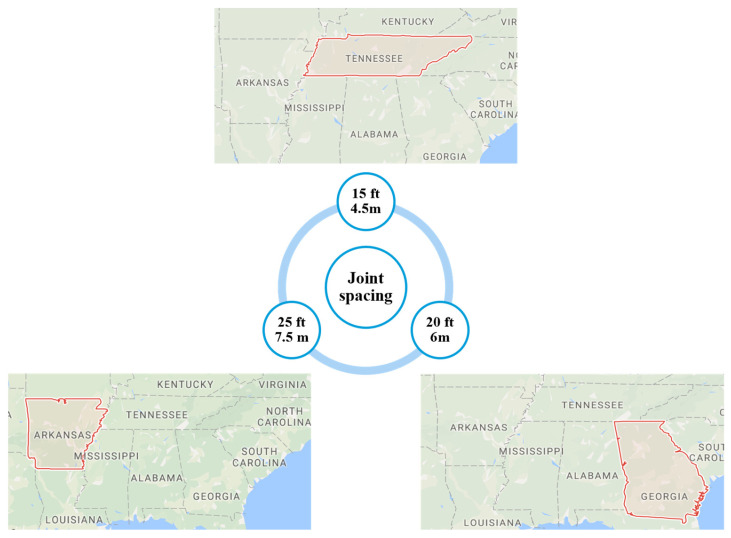
Locations of three extracted sections in terms of the LTPP database.

It is generally known that road performance is significantly affected by climate changes despite pavement types. To investigate the joint spacing effects in JPCP, three different survey sections with similar weather conditions were chosen, thus the weather influences could be generally eliminated. In the LTPP database, the road surface type was selected as JPCP. The climatic zone was set as Wet, Non-Freeze. In addition, the AADTT range was between 3000~6000. Based on the database, 46 sections were found that meet those defined conditions. After manually analyzing all 46 sections, 3 different sections with various critical joint spacings were determined. The number of lanes was 2 in each of the three sections. The sections were in Georgia, Tennessee, and Arkansas, respectively, and their corresponding joint spacings are summarized in Figure 1. The basic information for the three sections is also listed in Table 1.

Based on the aforementioned considerations, the three sections including 13-3016, 47-0601, and 05-0219 were then compare as shown in Table 2. The three sections had similar concrete slab thickness, functional class, number of lanes, and climate zone. All three sections had the LTPP data collected for more than 15 years, which is enough to accurately represent the long-term performance of the JPCP. In addition, dowel bars were involved in all of these sections at the joints.

**Figure 2:**
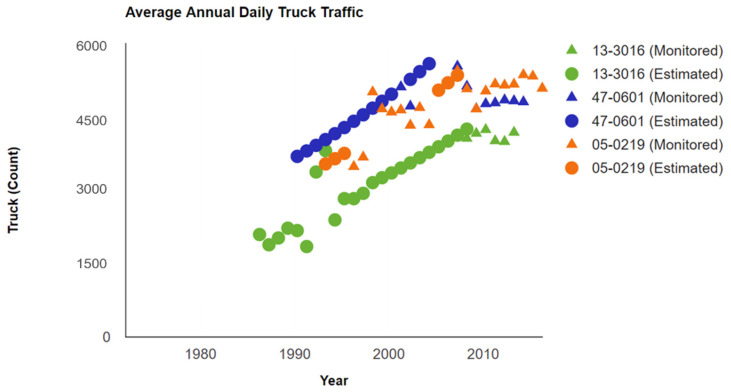
The change in average annual daily truck traffic (AADTT) in different sections.

The pavement performance was largely affected by the traffic volume. To eliminate the effect of traffic volume, based on the LTPP data, the changes in AADTT in the three different sections were compared as shown in Figure 2, which includes the estimated and monitored traffic data throughout the entire survey period. Obviously, the AADTT volumes increased during the service life, with the rapid development of economics and vehicles, and were all around 3000 to 6000 throughout the in-service period. The AADTT volume between 3000 and 6000 is usually defined as the medium traffic level, which can represent most of the traffic conditions in the field application of JPCP. Thus, the traffic volume for all selected sections was at the same level.

**Figure 3:**
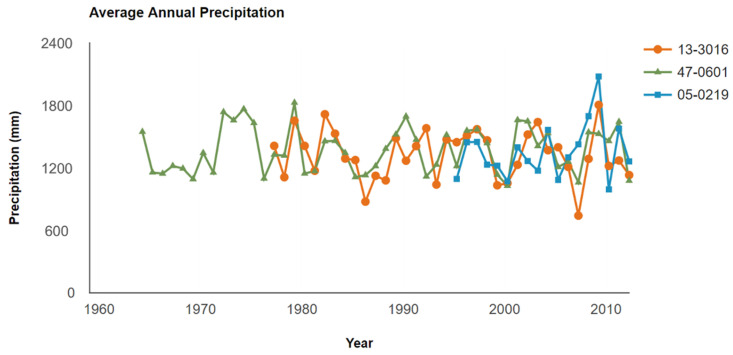
The change in precipitation in different sections.

The average annual precipitation at the three different locations was also compared as illustrated in Figure 3. It can be observed that the average annual precipitation in these three sections was mostly in the range of 1000 to 1600 mm during the survey periods. The maximum average annual precipitation of about 2100 mm was found in the 05-0219 section in the year 2010, while the lowest average annual precipitation of about 800 mm was found in the 13-3016 section in the year 2008. Overall, the precipitation conditions in these selected sections were kept consistent at a certain level, therefore the precipitation effect could be dismissed.

**Figure 4:**
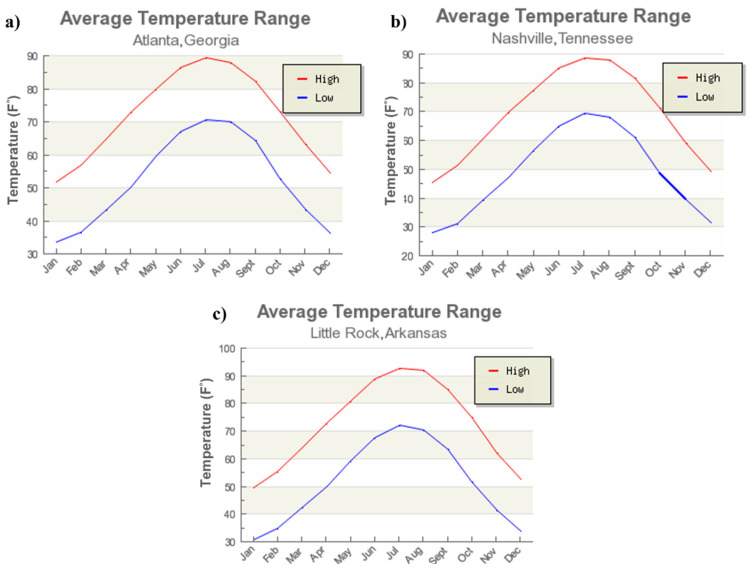
Average temperature changes: (**a**) Changes in section 13-3016; (**b**) Changes in section 47-0601; (**c**) Changes in section 05-0219.

As mentioned before, the temperature also influences the structure behaviors of JPCP to some extent. Therefore, not only the AADTT and precipitation have been considered, but also the temperature changes in these three sections have been compared. Figure 4 shows the average temperature changes through 12 months in sections 13-3016, 47-0601, and 05-0219, respectively. The temperature changes in the three different states showed the same trend and values, the lowest temperature was about 30 °F (−1.1 °C) and the highest temperature was about 90 °F (32.2 °C).

After comparing the basic structure design and environmental conditions in these sections, it can be predicted that the long-term performance of JPCP in these sections was mainly influenced by joint spacing changes. The transverse crack (count), average JPCP faulting, average IRI, and average FWD deflection in these three sections were compared to analyze the long-term pavement performance.

## 3. Results and Discussion

### 3.1. JPCP Transverse Cracking

**Figure 5:**
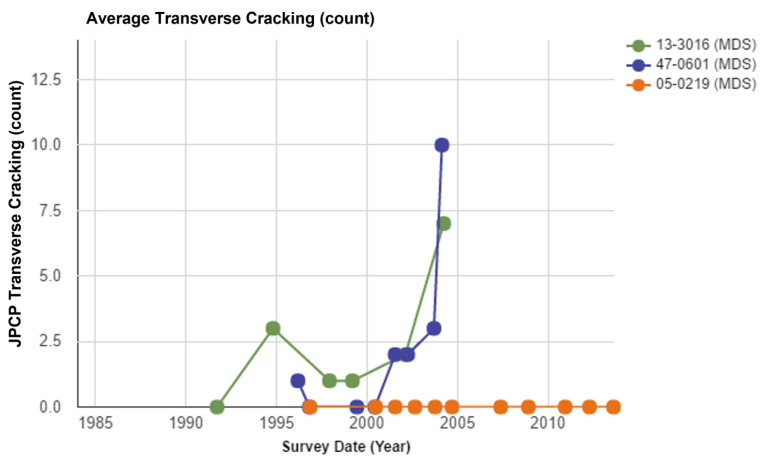
The comparison of the transverse cracking (count) with different joint spacings.

The JPCP transverse crack count was compared between three different joint spacings, as illustrated in Figure 5. From the results, it is obvious that section 0219 with the 15 ft joint spacing did not have any JPCP transverse cracks during the observation period. For sections 0601 (25 ft joint spacing) and 3016 (20 ft joint spacing), the transverse cracking volume was similar at first, but section 0601 had a higher number after 20 years of use. The total JPCP transverse cracking number was 13.6 in section 3016 and 18.2 in section 0601. The transverse cracking number increased with the increase in the in-service period. However, section 0601 had a higher accumulated number of cracks than section 3016. In terms of transverse cracking, the longer joint spacing was associated with a higher cracking volume.

Based on the mechanistic–empirical pavement design method, the maximum joint spacing can be calculated by Equation (1):

(1) $$l=\sqrt[{4}]{\frac{Eh^{3}}{12\left(1-\mu^{2}\right)k}}$$

where, l = radius of relative stiffness, *E* = modulus of elasticity of the concrete, *h* = slab thickness, *k* = modulus of subgrade reaction, and μ = Poisson’s ratio for concrete material.

With the determination of the radius of relative stiffness, the slab length is generally recommended to be in the range of 4l to 5l. By using plain concrete materials, the calculated spacing of joints is about 15 ft (4.5 m) for plain concrete. The LTPP extracted results were consistent with the traditional mechanistic–empirical calculation in terms of the optimum joint spacing for resistance to transverse cracking.

From the long-term performance analysis based on the LTPP data, it can be noticed that joint spacing is a considerable factor when designing a JPCP. To avoid transverse cracks and keep the pavement in good performance, joint contractions must be placed. Sometimes, dowel bars are also used to transfer load between concrete slabs. From the data comparison of different pavement performances, the behaviors of JPCP do show a relationship with the concrete slab joint spacing. According to the comparison, lower joint spacing (less than 20 ft) will support better long-term performance of the pavement than joint spacing which is higher than 25 ft.

### 3.2. Average JPCP Faulting

Faulting is a common distress type in JPCP and is defined as the difference in elevation across a transverse joint or crack. Faulting can result from a combination of factors such as inefficient load transfer at joints, slab pumping, slab settlements, curling, warping, and inadequate base support conditions. Faulting plays a predominant role in pavement surface roughness over time, affecting ride comfort and driver safety. Moreover, significant joint faulting has an adverse impact on pavement lifecycle costs for maintenance and rehabilitation as well as vehicle operating costs.

**Figure 6:**
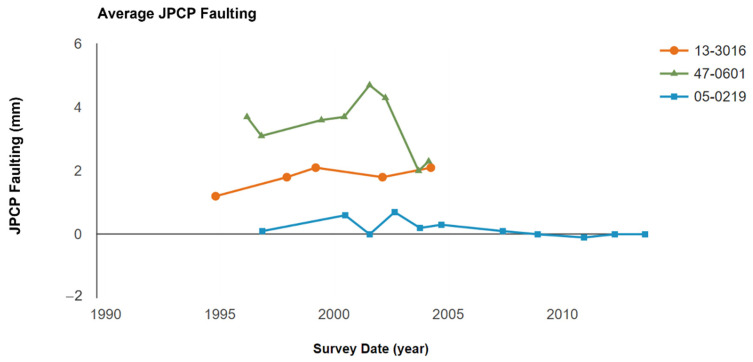
The comparison of the average JPCP faulting with different joint spacings.

According to the data from the LTPP database, the average JPCP faulting in these three sections showed significant differences. It is universally known that faulting is related to repeated load. From Figure 6, it is obvious that section 0601 (with 25 ft joint spacing) had the highest average JPCP faulting since the data began being collected. In contrast to this, section 0219 (with 15 ft joint spacing) had the lowest average JPCP faulting in the LTPP research period. Section 3016 (with 20 ft joint spacing) had a medium average JPCP faulting value. The average JPCP faulting depth in section 0601 (with 25 ft joint spacing) showed a reduction from the year 2000 to the year 2004. This could be attributed to differences and deviations in test methods. A previous study found that the JPCP faulting increased from 2 mm to 3 mm when the joint spacing increased from 15 ft to 20 ft [15], which is consistent with the results found in this study.

A Georgia fault meter (GFM) was used to collect joint and crack faulting data for the LTPP Program. The GFM is a manual faulting measurement (MFM) instrument that is time-consuming and inconvenient. The manual GFM uses a dial gauge to determine the positive or negative difference at a joint or crack, and the automated GFM uses the linear variable differential transformer (LVDT) to determine positive or negative faulting at a joint or crack. The legs of the GFM’s base are set on the slab in the direction of traffic on the left side of the joint. The joint must be centered between the guidelines shown on the side of the meter. The measuring probe contacts the slab on the approach side. Vertical movement of this probe is transmitted to an LVDT to measure joint faulting. Therefore, MFM data collection may contain some possible mistakes, due to equipment inaccuracy and operational error. In a word, even though LTPP data collection is a good data source, there are still some deviations within those data. In LTPP data collection methods, the faulting is determined by MFM, which is not accurate. Some more up-to-date ways can be used to detect faulting at a more accurate level.

However, the average JPCP faulting in these three different sections is different and has stratification. Section 0219, with 15 ft joint spacing, demonstrated the best performance among these three sections, suggesting that in the long-term service period, a lower joint spacing will contribute to lower JPCP faulting.

### 3.3. Average International Roughness Index

The international roughness index (IRI) is a scale for roughness based on the simulated response of a generic motor vehicle to the roughness in a single-wheel path of the road surface [26]. IRI is related to pavement flatness and driving comfort [27]. Ride comfort depends on human response to vibration, vehicle response to the road, and road roughness. The only factor which influences the ride comfort that is controlled by pavement is the road surface roughness. Therefore, IRI is a very important factor in considering long-term pavement performance.

**Figure 7:**
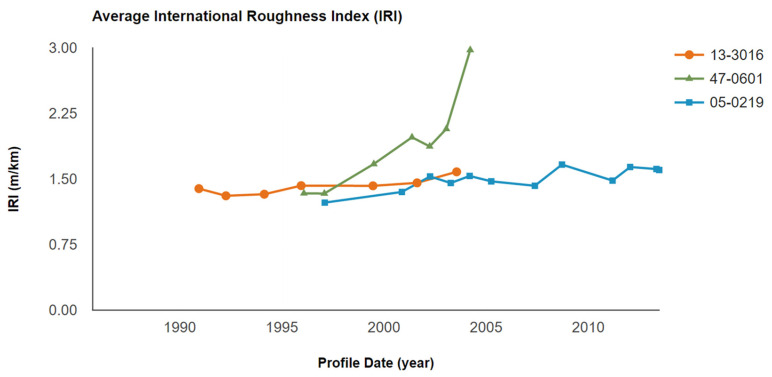
The comparison of the IRI with different joint spacings.

From LTPP data, the average IRI was collected. As can be seen in Figure 7, section 0219 (with 15 ft joint spacing) had the lowest IRI value consistently throughout the LTPP research period. Section 0219 had almost the same IRI value after construction but had some minor increases in the later years. For section 0601 (with 25 ft joint spacing), the IRI value was almost the same at first but then increased dramatically in the latter years, which meant the surface smoothness was significantly diminished during the increasing traffic volume and reduced durability. The IRI value showed a constant trend and was mostly lower than 1.5 m/km in section 3016 and section 0219. The lowest joint spacing of 15 ft contributed to the lowest and the most consistent IRI value (1.3 m/km) among the three different joint spacings, which was also in agreement with the findings in terms of the JPCP faulting

There are IRI categories given by the Federal Highway Administration (FHWA), shown in Table 3. An IRI value that is lower than 1.5 m/km means the pavement roughness is in good condition, and the acceptable IRI value should be lower than 2.7 m/km to maintain a workable surface roughness. Section 3016 (with 20 ft joint spacing) and section 0219 (with 15 ft joint spacing) fell into the good roughness category by this standard. However, section 0601 which had the highest joint spacing (25 ft) had an unacceptable value after being in service for ten years. According to those comparisons, joint spacing which is lower than 20 ft will contribute to good performance in long-term pavement smoothness and lower IRI values. Another study found that the IRI increased when the joint spacing changed from 12 ft to 20 ft, with a slab thickness that was greater than 7 in [28], which is also in agreement with the findings in this investigation.

Throughout the whole life cycle of the JPCP, maintenance is a considerable part of the economic cost during the reduction of IRI. The pavement with a higher IRI will result in a higher cost of maintenance. Therefore, it is important to determine an optimum joint spacing when designing a JPCP to diminish the maintenance costs.

### 3.4. Average FWD Deflection

**Figure 8:**
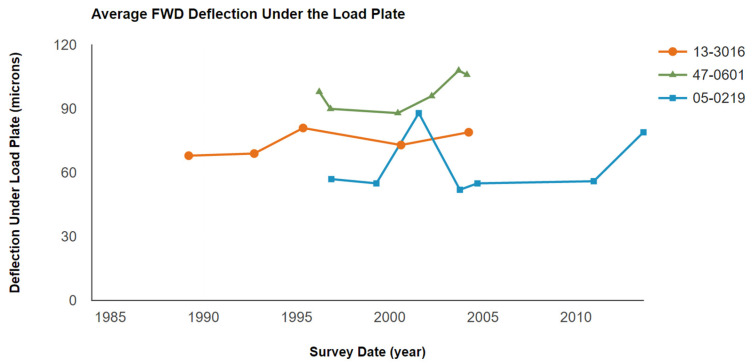
Average FWD deflection.

The FWD deflection can be used to investigate the dynamic deformation behavior of pavement structures. During the service period, the average FWD deflections changed continuously in the selected sections, as shown in Figure 8.

A comparison of the FWD deflections among the three sections shows that the FWD deflection was always higher in the 47-0601 section with a joint spacing of 25 ft, while the lowest FWD deflection was generally found in the 05-0219 section with a joint spacing of 15 ft. It is known that the lower the FWD deflection, the better the dynamic stability of the pavement structures. However, the trend of FWD deflection change was not constant in all sections. For instance, the FWD deflection first increased and then decreased in section 05-0219, in which an even higher FWD deflection measurement was found compared to that of section 13-3016 (with a joint spacing of 20 ft) around the year 2002. Consequently, even if the FWD deflection measurements can reflect the dynamic stability of the JPCP, it is not recommended to directly determine the status of the pavement with a single-year measurement since the FWD deflection measurements are considerably affected by other unexpected factors. It would be better to evaluate the FWD deflection with a long-term investigation, such as the LTPP method used in this study.

In this study, load transfer between adjacent concrete slabs was not considered. Aggregate interlocking between two slabs is the simplest way to transfer load. However, cracks will occur once the joints have been constructed, thus aggregate interlock performance within two slabs will be considerably reduced since dowel bars are placed in the joint contractions to help transfer load from one concrete slab to another. The long-term behaviors of dowel bars will also have an impact on the performance of JPCP. Moreover, sealed and unsealed joints are also factors that contribute to the JPCP long-term performance. In future studies, those factors could be investigated further to obtain a more all-around optimized JPCP performance-based design.

## 4. Conclusions

In this study, the effects of joint spacings on the long-term pavement performance in JPCP were investigated using the extracted LTPP data, collected for more than 15 years. With the increase in joint spacing, the development of transverse cracks was significantly increased, especially in the largest joint spacing of 25 ft, which showed 10 transverse cracks in later service life. With the change in joint spacings in JPCP, the dimension of the faulting was also changed, and decreased joint spacings could effectively control the development of faulting. The longer the joint spacing, the larger the IRI values in later service life, demonstrating worse surface roughness. The changing trend of FWD deflection was not consistent during the survey period, which is not critical to justify the long-term pavement performance of JPCP when compared with other involved performances.

In the future application of JPCP, the joint spacing should be designed to be lower than 20 ft to minimize the reduction in durability during the service life. An optimized joint spacing, considering the long-term pavement performance, is recommended to be 15 ft in most cases.

## Figures and Tables

**Table 1:** The locations and joint spacings of selected projects.

State Code	13	47	05
Section ID	3016	0601	0219
State/Province	Georgia	Tennessee	Arkansas
County	Haralson	Madison	Saline
Joint spacing (ft)	20 (6 m)	25 (7.5 m)	15 (4.5 m)

**Table 2:** The circumstances and physical conditions of selected sections.

Sections	13-3016	47-0601	05-0219
Route, Direction	Interstate–1, East Bound	Interstate–1, West Bound	Interstate–1, West Bound
GPS Lat, Long	33.68136, 85.29316	35.71699, 88.6383	34.51776, 92.68929
Functional Class	Rural Principal Arterial-Interstate	Rural Principal Arterial–Interstate	Rural Principal Arterial-Interstate
Number of Lanes	2	2	2
Concrete Slab Thickness (in.)	11.10 (28 cm)	9.00 (23 cm)	11.10 (28 cm)
Climatic Zone	Wet, Non-Freeze	Wet, Non-Freeze	Wet, Non-Freeze
Date of Construction	1 December 1977	1 June 1964	1 October 1995
Date Included in LTPP	1 January 1987	1 January 1987	1 September 1993
LTPP Monitoring Status (Data Inactive)	Out–of–study (15 June 2007)	Out–of–study (1 May 2004)	Out–of–study (20 August 2013)

**Table 3:** FHWA IRI Categories.

Roughness Category	IRI Value (m/km)
Good	<1.5
Acceptable	<2.7

## Data Availability

The data can be provided if needed by the corresponding author.
